# Optimizing Aerosol Jet Printing Process of Platinum Ink for High-Resolution Conductive Microstructures on Ceramic and Polymer Substrates

**DOI:** 10.3390/polym13060918

**Published:** 2021-03-16

**Authors:** Pavel V. Arsenov, Alexey A. Efimov, Victor V. Ivanov

**Affiliations:** Moscow Institute of Physics and Technology, National Research University, 141701 Dolgoprudny, Russia; efimov.aa@mipt.ru (A.A.E.); ivanov.vv@mipt.ru (V.V.I.)

**Keywords:** platinum nanoparticles, Pt nano-ink, aerosol jet printing, resistivity, polymer substrate

## Abstract

Printing nano-ink with platinum nanoparticles to generate conductive microstructures for electronics on different types of substrates has gained increasing interest in recent years. To solve the problem of the low conductivity of platinum (Pt) nano-ink, we synthesized chemically pure Pt nanoparticles with sizes of 18.2 ± 9.0 nm by spark discharge method. A low toxic solvent, ethylene glycol with water, was used to ensure the aggregation stability of Pt nanoparticles. Polyvinylpyrrolidone was used as an adhesive additive and binder in the nano-ink. Narrow and conductive Pt lines were generated by aerosol jet printing technology. The resistivity of the Pt lines sintered at 750 °C on alumina substrate was found to exceed the bulk Pt by about 13%. Moreover, the Pt film fabricated on polymer substrates has demonstrated excellent mechanical flexibility in terms of twisting tests.

## 1. Introduction

In recent years, printing processes have gained interest as a means of making cost-effective electronic circuits and devices [1,2]. The most known traditional printing processes are lithography, etching, and sputtering [3,4]. However, the limited choice of substrate materials, the high cost, and the time-consuming manufacture of electronic devices led to the development of technologies such as screen printing, inkjet printing, gravure printing, and aerosol jet printing [5,6,7,8]. These fast production technologies are an excellent solution for the manufacturing of organic light-emitting diodes, sensors, displays, and thin-film transistors [9,10,11,12]. Functional nano-ink in the form of colloidal suspensions of nanoparticles in various solvents is a key component for these technologies [13,14,15]. One of the main problems of additive technologies is the development of suitable inks. The composition of the nano-ink determines not only compatibility with the printer’s spray system but also the quality of the printed films and microstructures [16,17,18,19]. In turn, the processes of manufacturing electronic devices imposed many requirements for printing resolution. In order to form a high density of elements in the circuit, it is necessary to ensure the minimum size of structures in the range from 10 to 30 microns [16,20]. From this point of view, aerosol jet printing is the most promising method, since it allows the formation of functional microstructures with lateral dimensions of the order of 10 μm on various substrate materials, including flexible polymer substrates [21,22,23,24].

Today, platinum (Pt) nanoparticles are in demand in printed electronics [25,26,27]. Due to their remarkable catalytic activity, they are widely used as material for high corrosion resistance contacts, thermal, gas, and biological sensors [26,28,29,30]. The chemical reduction method is widely known for the preparation of nano-ink based on nanoparticles [23]. However, nanoparticles synthesized by this method have surface contamination, which negatively affects the conductivity of the obtained ink [23,31]. Moreover, the resulting nanoparticles have a wide range of size distribution, while monodisperse metal nanoparticles are critical for obtaining a highly conductive nano-ink. In this article, we used platinum nanoparticles synthesized in a spark discharge [32] under air. This method of synthesis made it possible to obtain chemically pure nanoparticles [33] in order to prepare highly conductive nano-ink for aerosol jet printing.

The purpose of this work was to optimize the composition of platinum nano-ink for aerosol jet printing, namely, to select the concentrations of solvent and polymer as a binder to achieve a high quality of the formed microstructures. In the aerosol jet printing process, platinum lines and films were fabricated on rigid ceramic and flexible polymer substrates, respectively. We focused on investigating the effect of aerosol jet printing and sintering process parameters on the geometry and resistivity of printed platinum lines. Flexible polymer substrates were used to form conductive contacts at sintering temperatures lowered to 300 °C.

## 2. Materials and Methods

### 2.1. Synthesis of PtNPs and Formulation of Nano-Ink

**Figure 1:**
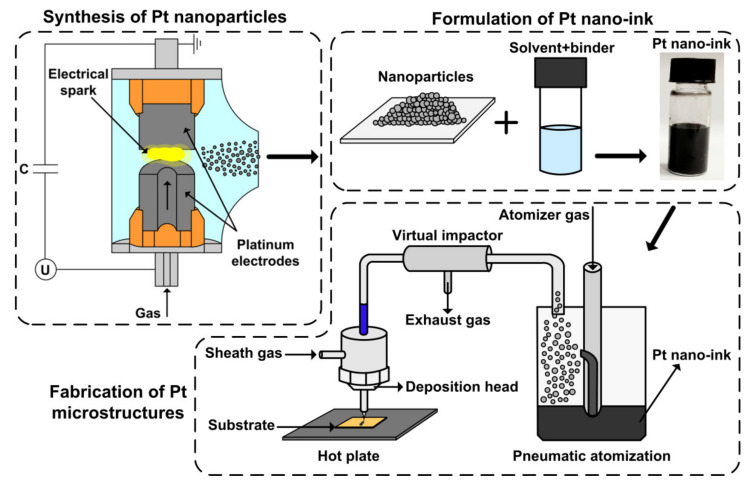
Schematic presentations: synthesis of platinum nanoparticles (PtNPs), formulation of Pt nano-ink, and fabrication of Pt microstructures using aerosol jet printing system.

An early developed and investigated spark discharge generator (SDG) [33] was used to synthesize platinum nanoparticles (PtNPs). PtNPs were obtained in the process of electrical erosion of platinum electrodes with a mass content of platinum of about 99.97%. The SDG consisted of a voltage source, a capacitor, and a chamber for collecting nanoparticles. A capacitor C = 107 nF was charged to a voltage of U = 2.3 kV, at which the self-breakdown of the gap between electrodes occurred, initiating an electrical spark. Filtered air at a pressure of 2 bars was supplied to the chamber, blowing out the nanoparticles. Then, PtNPs were collected on a capsule filter (TSI Inc., Shoreview, MN, USA) for the preparation of nano-ink and the following application in the aerosol jet printing (AJP) system (see Figure 1).

Fundamentally, a colloidal ink is composed of nanoparticles, dispersed in a vehicle representing a solvent with dissolved additives. In this paper, the basic principles for formulation of Pt nano-ink were based on the following requirements. First, the concentration of platinum in nano-ink should be over 20 wt. % in order to enable the fabrication of conductive microstructures on ceramic and polymer substrates at a moderate dosage of deposited ink, thus accelerating the production process. Second, the ink should keep relatively high colloidal stability to ensure sufficient reproducibility of the parameters of the printed microstructures. The third principle was to use solvents having moderate boiling points from 100 to 200 °C and low toxicities, which was significant in the ink application in aerosol jet printing technology. Finally, the ink should contain polymer as an additive for high adhesive strength and mechanical flexibility of printed microstructures.

The collected PtNPs were dispersed for 90 min in a mixture of ethylene glycol (EG) and water, which was used as a solvent for the nano-ink. EG was chosen as a non-volatile and low toxic solvent. Polyvinylpyrrolidone (PVP) was chosen as an adhesive additive and binder in nano-ink. It one of the most commonly used metallic nanoparticle surfactants, stabilizers, and adhesive additive in the ink formulation industry, because of its excellent solubility in ethylene glycol [34]. Ultrasonic treatment with a specific power of about 3 W/cm^3^ for 1 h was used to deagglomerate particles in nano-ink. The coarse fraction of particles in the ink was further removed from the nano-ink by sedimentation in a gravitational field for 24 h. The percentage mass concentration of EG with water, PVP, and PtNPs were selected so that the surface tension and viscosity of the nano-ink were compatible with AJP technology.

### 2.2. AJP Testing of Prepared Pt Nano-Ink

The prepared nano-ink was tested with a commercial aerosol jet printer (AJ 15XE, Neotech AMT GmbH, Nuremberg, Germany). Nano-ink was sprayed with a pneumatic atomizer and focused on the substrate through a micro nozzle (see Figure 1). Ceramic alumina and flexible polyimide materials were used as substrates for AJP. Experiments on the study of AJP with Pt nano-ink were carried out with the purpose of forming narrow (up to 30 μm), homogeneous, and highly conductive platinum lines. For this, the parameters of AJP were optimized, such as the nozzle diameter *D*_n_, the aerosol *Q*_a_ and sheath *Q*_sh_ flow rates (in particular *FR* = *Q*_sh_/*Q*_a_), the substrate temperature *T*_s_ during the printing process, the substrate speed *V*_s_, and the number of printing layers *N*_p_. The investigated parameters of AJP are presented in Table 1.

After deposition, the Pt lines were sintered at a temperature of 500–750 °C in a muffle furnace in an air atmosphere for 2 h.

### 2.3. Characterization and Measurement

The morphology and size of the synthesized PtNPs were investigated using a scanning electron microscope (SEM) (JSM-7001F, JEOL Ltd., Tokyo, Japan) and a transmission electron microscope (TEM) (JEM-2100, JEOL Ltd., Tokyo, Japan). The elemental composition of the PtNPs was determined using energy-dispersive X-ray spectroscopy (EDX) in SEM. The surface tension and viscosity of the nano-ink were determined using an optical tensiometer (DSA25S, Krüss GmbH, Hamburg, Germany) and a viscometer (SV-10, A&D Company, Limited, Tokyo, Japan), respectively.

The width and microstructure of the formed Pt lines were investigated using an optical microscope (VHX-1000, KEYENCE, Itasca, IL, USA) and SEM. The resistivity of the sintered lines was calculated using the following Equation (1):

(1) $$\rho =\frac{R\cdot a}{l}$$

where *R*—electrical resistance; *a*—cross-sectional area of the printed line; *l*—length of the printed line.

The electrical resistance of the line *R* was measured using a 4-point method using a multimeter (U1253B, Agilent Technologies Inc., Santa Clara, CA, USA) and a precision current source (SourceMeter 2401, Tektronix Inc., Beaverton, OR, USA). The cross-sectional area *a* was measured using an optical 3D profilometer (S neox, Sensofar, Terrassa, Spain). The length *l* of the printed line was measured by optical microscope (VHX-1000, KEYENCE, Itasca, IL, USA).

## 3. Results and Discussion

**Figure 2:**
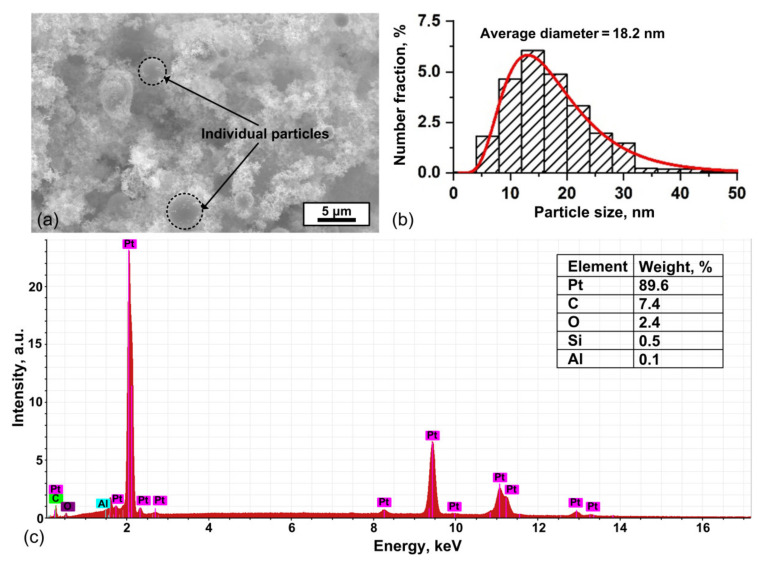
(**a**) SEM image, (**b**) histogram of the particle size distribution, and (**c**) typical EDX spectrum of PtNPs.

Figure 2a,b shows the SEM image and histogram of the particle size distribution for primary nanoparticles and aggregates, measured with a TEM. The particle size distribution histogram is well approximated by a log-normal function. According to the results of TEM analysis, it was determined that the average sizes of primary nanoparticles are 18.2 ± 9.0 nm. It was also established from the SEM results that the synthesized material also contains large individual particles up to 4 μm in size. Such large particles are formed as a result of splashing molten electrode material [35]. A typical elemental analysis of particles according to EDX spectrum is shown in Figure 2c. The oxygen and aluminum lines were due to the contribution of the ceramic alumina substrate.

In this paper, nano-ink was prepared from synthesized PtNPs using ethylene glycol (EG) and water as a solvent and a polyvinylpyrrolidone (PVP) as a binder. Their mass concentrations were determined in such a way that the values of surface tension and viscosity of nano-ink were compatible with aerosol jet printing technology [36].

The nano-ink was characterized by mass concentrations of EG with water, PVP, and PtNPs equal to 71 wt.%, 4 wt.%, and 25 wt.%, respectively. The achieved values of surface tension and viscosity of the optimized nano-ink were approximately 44 mN/m and 11 cP, respectively.

**Figure 3:**
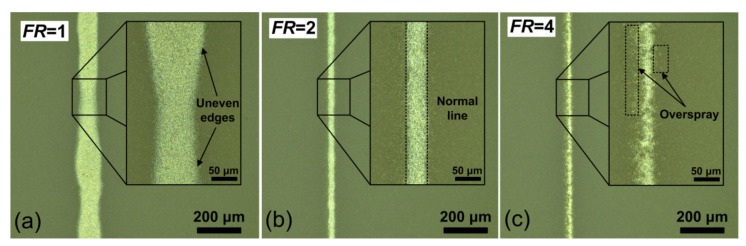
Optical images of platinum lines formed at different values of focusing ratio (**a**) *FR* = 1, (**b**) *FR* = 2, (**c**) *FR* = 4, and inserts with higher magnifications.

In order to form narrow (about 30 μm) lines, nozzles with diameters of 100, 150, and 300 μm were investigated. A nozzle with a diameter of 100 µm—due to a too narrow outlet orifice—often clogs. This nozzle is not suitable for continuous printing. The nozzle with *D*_n_ = 300 µm does not allow for obtaining lines with a width *w* of the order of 30 µm at the investigated values of *Q*_a_ and *Q*_sh_. In this regard, a nozzle with *D*_n_ = 150 μm was used in the experiments. Using this nozzle, the influence of the focusing ratio *FR* = *Q*_sh_/*Q*_a_ on the width *w* and the homogeneity of the obtained lines was established, see Figure 3.

Figure 3a shows that at the value of *FR* = 1, the line has uneven edges and an average value of *w* about 110 μm. At too high values of *FR* = 4 (see Figure 3c), the line turns out to be discontinuous with high roughness and overspray effect. So, the optimum value is *FR* = 2, where a homogeneous normal line with a small width value of *w* about 30 μm is obtained (see Figure 3b). The corresponding optimum values of *Q*_a_ and *Q*_sh_ were 10 and 20 sccm, respectively.

**Figure 4:**
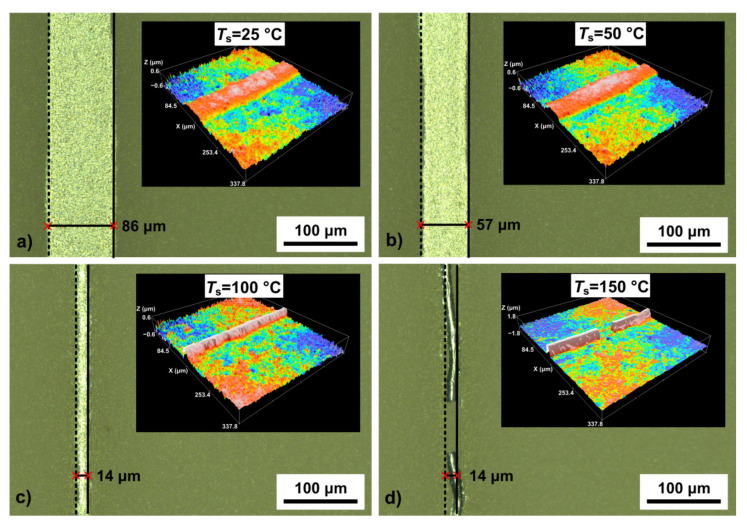
Optical images and profiles of platinum line (on inserts) formed at different values of substrate temperature (**a**) *T*_s_ = 25 °C, (**b**) *T*_s_ = 50 °C, (**c**) *T*_s_ = 100 °C and (**d**) *T*_s_ = 150 °C, at value of *FR* = 2.

The spreading of Pt nano-ink during the printing process was controlled by the substrate temperature *T*_s_. Figure 4 shows optical images and profiles (on inserts) of platinum lines formed at different values of *T*_s_ in a range from 25 to 150 °C.

**Figure 5:**
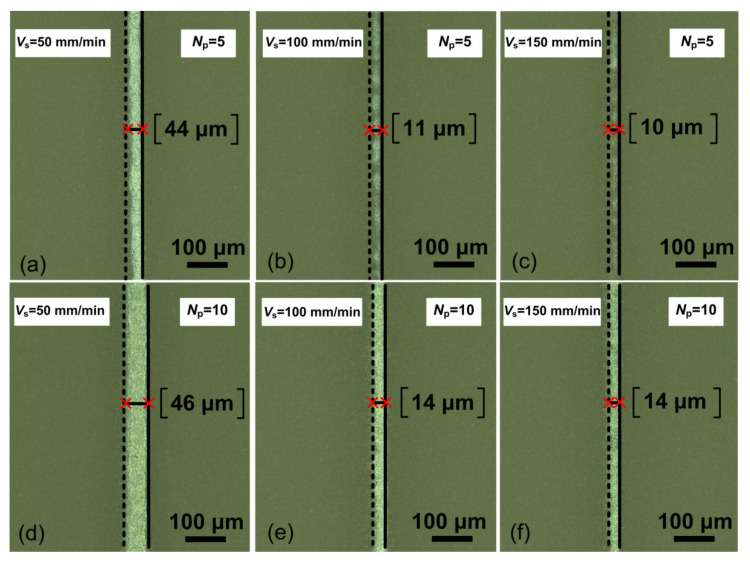
Optical images of platinum lines formed at different values of the substrate speed and the number of print layers (**a**) *V*_s_ = 50 mm/min and *N*_p_ = 5, (**b**) *V*_s_ = 100 mm/min and *N*_p_ = 5, (**c**) *V*_s_ = 150 mm/min and *N*_p_ = 5, (**d**) *V*_s_ = 50 mm/min and *N*_p_ = 10, (**e**) *V*_s_ = 100 mm/min and *N*_p_ = 10, (**f**) *V*_s_ = 150 mm/min and *N*_p_ = 10, at values of *FR* = 2 and *T*_s_ = 100 °C.

Figure 4 shows that the value of *w* significantly decreases from 86 to 14 μm with an increase in *T*_s_ from 25 to 150 °C, respectively. From the profilometry data, it can be seen that the line heights also increase, which, in particular, leads to an increase in the aspect ratio of the lines. This increase is due to the contact angle between microdroplets, which increases with substrate temperature [37,38]. Thus, it has been found that the optimum value of *T*_s_ during printing should be in the order of 100 °C. At this substrate temperature, we carried out the experiments to establish the effect of the substrate speed *V*_s_ and the number of printing layers *N*_p_ on the width *w* and homogeneity of the obtained lines. Figure 5 shows optical images of platinum lines formed at different values of *V*_s_ and *N*_p_ at *T*_s_ = 100 °C and *FR* = 2.

Figure 5 shows that the optimum value of *N*_p_ is 10 layers. Figure 5a,b,c shows that at *N*_p_ = 5 and different values of *V*_s_, the line is discontinuous with insufficient thickness for enough conductivity. In this case, the optimum value of *V*_s_ = 100 mm/min, since the line width *w* is less than 30 μm and has a homogeneous structure in comparison with other values of *V*_s_ (see Figure 5d–f). As a result of the research, the processes of the formation of conductive and narrow Pt microstructures were optimized. The optimized parameters of AJP while forming single narrow lines (about 30 μm) are presented in Table 2.

**Figure 6:**
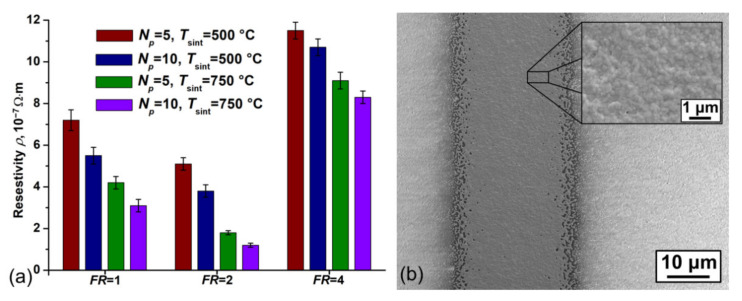
(**a**) Dependence of the resistivity of platinum lines on the values of *FR*, *N*_p_, and *T*_sint_ and (**b**) typical SEM images of the surface of the sintered line, printed with optimized parameters.

Figure 6a shows the dependence of the resistivity of platinum lines on the values of *FR*, *N*_p_, and the sintering temperature *T*_sint_. The minimum value of *ρ* on the alumina substrate was (1.2 ± 0.1) × 10^−7^ Ω·m and was achieved with the values of the optimized parameters presented in Table 2 and *T*_sint_ = 750 °C. The achieved value of *ρ* is approximately 13% higher than the resistivity of bulk platinum 1.06 × 10^−7^ Ω·m. This result is achieved due to the low residual porosity of the sintered material at the value of *T*_sint_ = 750 °C (see Figure 6b). Table 3 presented a comparison of the resistivity with the other data of the state-of-the-art methods for printing technologies with prepared Pt nano-ink.

**Figure 7:**
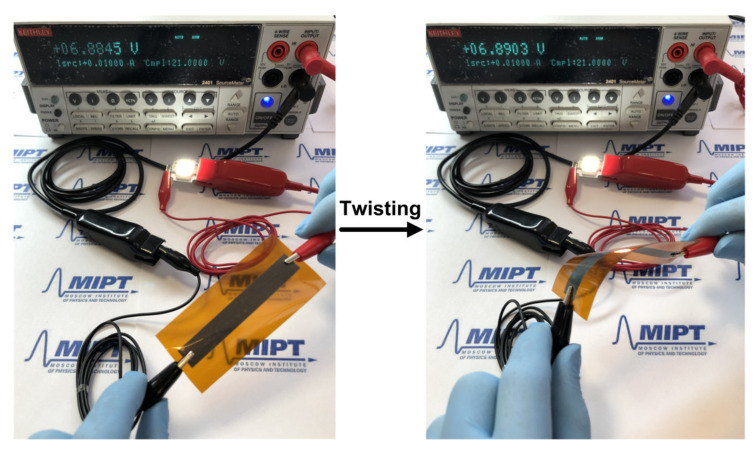
Photographs of the LED circuit with the printed Pt film on flexible polyimide substrate during the twisting tests.

To demonstrate the application of prepared platinum nano-ink on flexible polyimide substrates, a film with sides of 10 and 70 mm was formed. A platinum film was sintered at 300 °C and used as a conductive wire to illuminate a light-emitting diode (LED) (COB D506, Guangdong, China) during twisting tests (see Figure 7). The applied current and voltage were 0.01 A and 6.89 V, respectively. The voltage changes were measured to evaluate the effect of twisting tests on the resistance of Pt film with a thickness of 5 μm, printed on a flexible polyimide substrate.

The voltage values vary within 0.006 V, suggesting that the change in resistance of the printed Pt film is less than 0.6 Ω. The illumination intensity of the LED remains constant without deterioration after twisting. These results show that prepared ink has good adhesive strength and can be used in flexible conductive electronic devices.

## 4. Conclusions

Platinum nanoparticles obtained by the spark discharge method can be used to create a highly conductive functional nano-ink. A low toxic solvent, ethylene glycol with water, is used to ensure the aggregation stability of nanoparticles. Polyvinylpyrrolidone is used as an adhesive additive and binder in the Pt nano-ink. The ink is characterized by the following basic parameters for compatibility with aerosol jet printing system: platinum nanoparticles concentration 25 wt%, viscosity 11 cP, and surface tension 44 mN/m. The prepared Pt nano-ink is an excellent solution for forming narrow (up to 30 microns) and highly conductive platinum lines using an aerosol jet printing system. The resistivity of the Pt lines sintered at 750 °C is (1.2 ± 0.1) × 10^−7^ Ω·m, which exceeds bulk Pt by about 13%. Thin Pt film was formed on flexible polymer substrates at a sintering temperature of 300 °C to demonstrate the application of prepared Pt nano-ink. The light intensity of the LED circuit, connected to Pt film, remains constant during twisting tests. These results demonstrate that the printed platinum microstructures also have good adhesive strength and mechanical flexibility. Thus, the prepared platinum nano-ink can be promising not only for the manufacture of printed electronics on thermally sensitive flexible polymer substrates but also for highly conductive structures with application in thermal and gas sensors.

## Figures and Tables

**Table 1:** Investigated parameters of aerosol jet printing (AJP) during the formation of Pt microstructures.

*D*_n_, μm	*Q*_a_, sccm	*Q*_sh_, sccm	*T*_s_, °C	*V*_s_, mm/min	*N*_p_
100–300	10–40	15–50	25–150	50–150	5–10

**Table 2:** Optimized parameters of AJP during the formation of narrow (about 30 μm) Pt lines.

*D*_n_, μm	*Q*_a_, sccm	*Q*_sh_, sccm	*T*_s_, °C	*V*_s_, mm/min	*N*_p_
150	10	20	100	100	10

**Table 3:** Comparison of the resistivity of platinum structures and parameters of Pt nano-ink.

Reference	Printing Method	Particle Size, nm	Solid Content, wt%	Solvent	Type of Substrate	Resistivity *ρ*, 10^−7^ Ω·m
Schubert et al., 2018 [27]	Inkjet printing	<200	20	Water-based	Alumina	60
Zea et al., 2019 [39]	Inkjet printing	25–100	20.5	Ethylene glycol and water	PEN	6.3
Vechembre et al., 2001 [40]	Screen-printing	-	65	Organic	Alumina	1.6
Vasiliev et al., 2015 [41]	Aerosol jet printing	10–30	15–20	Organic	Alumina	30
This work	Aerosol jet printing	18.2	25	Ethylene glycol and water	Alumina	1.2

## Data Availability

The data presented in this study are available on request from the corresponding author.
